# Efficacy and safety of ivermectin–albendazole combination versus ivermectin or albendazole monotherapy in soil-transmitted helminthiasis: A systematic review and meta-analysis

**DOI:** 10.1097/MD.0000000000041280

**Published:** 2025-01-17

**Authors:** Hurais Malik, Muhammad Fazal Ud Din, Muhammad Aqib Faizan, Tooba Rehman, Muhammad Hudaib, Syed Muhammad Abdullah Shah, Abdullah Abid Khan, Syeda Fatima Amir, Musarrat Fraz, Maham Khalid, Muhammad Umair Anjum, Muhammad Omar Larik, Pratik Bhattarai

**Affiliations:** a Department of Medicine, Fazaia Ruth Pfau Medical College, Karachi, Pakistan; b Primary and Secondary Healthcare Department, Punjab, Pakistan; c Department of Medicine, Gomal Medical College, Khyber Medical University, Pakistan; d Department of Medicine, Peoples University of Medical and Health Sciences, Nawabshah, Pakistan; e Department of Medicine, Dow Medical College, Karachi, Pakistan; f Department of Medicine, Dow International Medical College, Karachi, Pakistan; g Department of Medicine, Manipal College of Medical Sciences, Pokhara, Nepal.

**Keywords:** albendazole, efficacy, ivermectin, meta-analysis, safety, soil-transmitted helminths

## Abstract

**Background:**

Soil-transmitted helminthiasis remains a daunting challenge to global health, exerting its greatest toll on resource-limited regions of the world. A dual drug approach using the co-administration of ivermectin and albendazole has shown promising results in comparison to the traditional monotherapy strategy. In light of this, a systematic review and meta-analysis of randomized controlled trials was conducted.

**Methods:**

Several electronic databases including PubMed, Cochrane Central, Google Scholar, and Embase were explored to search for relevant studies from inception to September 2023. The Cochrane Risk of Bias Tool for Randomized Controlled Studies was utilized to evaluate the quality of studies.

**Results:**

A total of 8 randomized controlled trials, reporting 10 patient populations, were included. The treatment of trichuriasis significantly favored the dual therapy regimen of ivermectin–albendazole over albendazole-only monotherapy (risk ratio [RR]: 2.86; 95% confidence interval [CI]: 1.66–4.93; *P* = .0002), with no significant differences observed for ascariasis and hookworm. The treatment of trichuriasis and hookworm significantly favored the dual therapy regimen of ivermectin–albendazole over ivermectin-only monotherapy (RR: 1.86; 95% CI: 1.56–2.21; *P* < .00001 and RR: 2.31; 95% CI: 1.23–4.31; *P* = .009, respectively). There were no statistically significant differences between dual therapy and monotherapy in terms of adverse effects.

**Conclusion:**

These findings highlight the nuanced effectiveness of combined therapy specific to certain helminth types, in addition to their comparable safety profiles, thereby providing pivotal insights that contribute to the evolving landscape of soil-transmitted helminth treatment strategies.

## 1. Background

Soil-transmitted helminthiasis (STH) remains a daunting challenge to global health, exerting its greatest toll on populations residing in resource-limited regions characterized by inadequate sanitation and hygiene practices. Within this group of parasitic infections, we encounter notorious pathogens such as *Ascaris lumbricoides*, *Trichuris trichiura*, and the following hookworms: *Necator americanus* and *Ancylostoma duodenale*.^[1]^ Collectively, STH infections have cast their shadow over nearly 1.5 billion individuals worldwide, imposing a profound health burden that cannot be neglected.^[2]^ Among its primary victims are children, whose growth, cognitive development, and overall well-being are subject to risk of grave compromise.^[3]^ Furthermore, STH inflicts debilitating symptoms, including anemia, diarrhea, and abdominal pain, while also indirectly heightening the susceptibility to other parasitic infections.^[4]^

To address this pressing issue of global health, the World Health Organization (WHO) has set ambitious targets aiming to reduce the prevalence of STH through preventive chemotherapy programs. These programs hinge on the administration of a single dose of either albendazole or mebendazole, the 2 preferred agents of the benzimidazole group, and primarily target preschool and school-going children in populations where STH prevalence exceeds 20%.^[5]^ Albendazole and mebendazole, traditional anthelmintics, have long served as the cornerstone therapies for STH infections. However, their efficacy against certain STH infections remains less than satisfactory.^[6]^ As a result, ongoing research endeavors have delved into alternative drug regimens, including combination therapies, in a quest to address potential drug resistance and enhance cure rates (CRs).^[7]^

A particularly promising alternative to traditional monotherapy is the dual-drug approach, which has demonstrated a desirable capacity to achieve higher CRs and sustained reductions in STH infection prevalence, particularly against the most frequent STH species: *A. lumbricoides*, *T. trichiura*, and hookworms.^[8,9]^ Notably, the co-administration of albendazole and ivermectin has emerged as an attractive treatment regimen, boasting favorable tolerability and a commendable safety profile.^[10]^ While the combination of ivermectin and albendazole is well-established for its effectiveness in treating lymphatic filariasis,^[8]^ its efficacy and safety profile specifically in the context of STH infections warrant further comprehensive evaluation.

Amidst the relentless endeavors of the global health community to discover effective and sustainable solutions against STH, there is an emerging necessity to comprehensively assess the existing body of evidence concerning the efficacy and safety of co-administration of ivermectin and albendazole. While previous research has offered valuable insights, the contemporary landscape demands an inclusive systematic review and meta-analysis, equipped with the capacity to quantify the superiority of combined therapy in opposition to monotherapy, and pave the path for informed and targeted interventions, ultimately bringing us closer to the goal of liberating vulnerable populations from STH.

## 2. Methodology

### 2.1. Data sources and search strategy

This systematic review and meta-analysis followed the guidelines established by the “Preferred Reporting Items for Systematic Reviews and Meta-Analyses”.^[11]^ We conducted a comprehensive search, from inception to September 2023, across 4 prominent databases, including (i) PubMed, (ii) Cochrane Central, (iii) Google Scholar, and (iv) Embase. To identify pertinent articles, we employed a robust search strategy combining keywords and Medical Subject Heading (MeSH) terms, including: “soil-transmitted helminths,” “STH,” “helminths,” “Aschelminthes,” “Nematomorpha,” “Ascaris lumbricoides,” “Whipworm,” “Trichuris trichiura,” “Hookworm Infection,” “Ivermectin,” and “Albendazole.” The complete search strategy utilized for each database is available in Table S1, Supplemental Digital Content, http://links.lww.com/MD/O278.

### 2.2. Eligibility criteria

All studies conforming to the following eligibility criteria were eligible for inclusion, including: (i) double-arm randomized controlled trials, (ii) study populations with at least one diagnosed STH infection, including *A lumbricoides*, *T trichiura*, *N americanus*, and *A duodenale*, (iii) intervention of monotherapy using albendazole or ivermectin, and combination therapy using ivermectin and albendazole, (iv) studies employing specific dosages of each drug, including 200 µg or 600 µg for ivermectin, and 400 mg for albendazole, and (v) studies reporting at least one outcome of interest. Excluded studies were those published in languages other than English, as well as review articles, case reports, case series, cross-sectional studies, editorials, commentaries, and animal studies.

### 2.3. Data extraction and quality assessment

After completing our thorough systematic search, all potentially relevant articles were exported to EndNote Reference Library (Version X7.5; Clarivate Analytics, Philadelphia, PA) in order to identify and eliminate duplicate studies. Two authors independently screened titles and abstracts, removing irrelevant articles that did not comply with the eligibility criteria. Subsequently, a full-text review was conducted to assess eligibility. Any discrepancies arising during this process were resolved through consultation with a third author. The following baseline characteristics were extracted from the list of included studies: (i) age of population, (ii) number of participants in each cohort, (iii) sex of population, (iv) height and weight, and (v) parasite being treated. Additionally, the infection intensity was also extracted, classified into light, moderate, or heavy in accordance to WHO guidelines.^[12]^

The primary efficacy outcomes for our analysis included CR and egg reduction rate (ERR). Furthermore, we evaluated secondary safety outcomes, including (i) total number of adverse events, (ii) incidence of abdominal pain, (iii) incidence of fever, (iv) incidence of body rash, (v) incidence of itching (pruritus or urticaria), (vi) incidence of headache, (vii) incidence of vomiting, and (viii) incidence of nausea.

The assessment of quality and risk of bias was evaluated using the Cochrane Risk of Bias tool^[13]^ via 2 independent reviewers, with any discrepancies resolved by a third reviewer. The studies were evaluated in accordance with their randomization process, deviations from intended interventions, missing outcome data, measurement of the outcome, and selection bias within the reported results. Subsequently, the studies were declared as “low risk” of bias, “moderate” risk of bias, or “high” risk of bias.

### 2.4. Statistical analysis

All statistical analyses were performed using RevMan (version 5.4.1; Copenhagen: The Nordic Cochrane Centre, The Cochrane Collaboration, 2020). All dichotomous outcomes were aggregated using risk ratios (RR), along with their 95% confidence interval (CI) values. A random-effects model was utilized to account for heterogeneity among studies. In studies with low levels of heterogeneity, the fixed-effects model was employed. Results were considered statistically significant when the *P*-value was ≤.05. Higgins *I*^2^ statistics were used to report heterogeneity for each outcome, classifying it as low (*I*^2^ < 50%), moderate (*I*^2^ = 50–75%), or high (*I*^2^ > 75%).^[14]^

## 3. Results

### 3.1. Literature search and study selection

**Figure 1.**
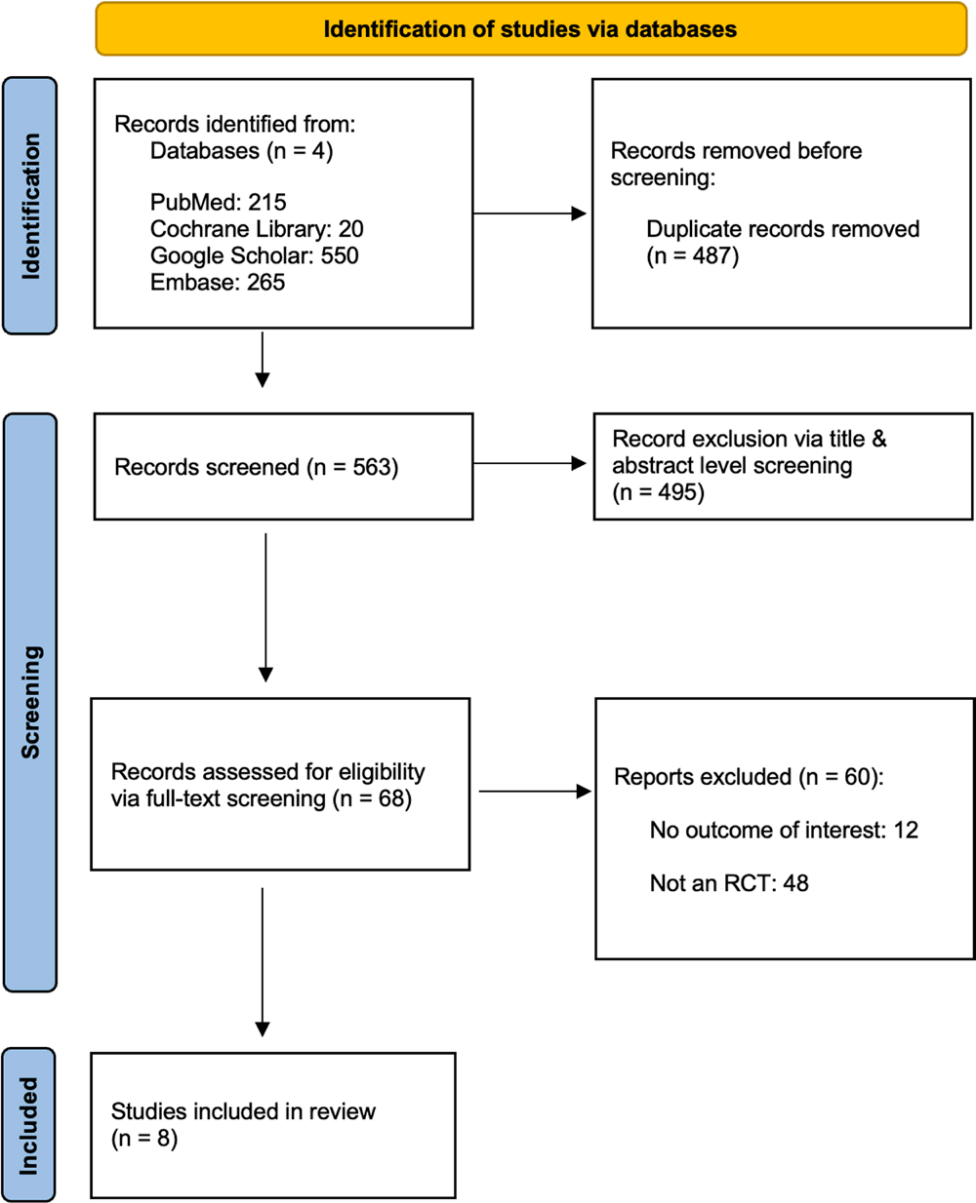
PRISMA flowchart illustrating the systematic screening process.

The thorough screening through the 4 included databases including: (i) PubMed, (ii) Cochrane Central, (iii) Google Scholar, and (iv) Embase, yielded a total of 1050 articles. After the exclusion of duplicate records, there were 563 studies subject to title-and-abstract level screening by independent investigators. Subsequently, there were 68 studies subject to full-text review. Ultimately, there were 8 studies declared as eligible for inclusion within this qualitative and quantitative synthesis.^[10,15–21]^ The study selection process is illustrated in the Preferred Reporting Items for Systematic Reviews and Meta-Analyses flowchart in Figure 1.

### 3.2. Study characteristics and risk of bias assessment

Out of the 8 selected studies, 5 included children ranging from 2 to 14 years of age, whereas one study included both adults and children older than 6 years of age. One study included pregnant females of any parity attending antenatal-clinic in their second trimester. All included trials used the same dose of albendazole (400 mg) and ivermectin (200–600 µg/kg). The efficacy outcomes reported in all studies were assessed between 7- and 21-days posttreatment, with the exception of Beach et al who assessed the primary outcome after 4 months posttreatment.^[16]^ The study population baseline characteristics are available in Table 1.

**Table 1:** Study characteristics and patient baseline characteristics of the included study population.

Publication	Mean treatment duration	Mean age, years (SD)			Male, n			Height (cm)			Weight (kg)			Intensity of infection, n (ALB + IVM/ALB/IVM)				
		ALB + IVM	ALB	IVM	ALB + IVM	ALB	IVM	ALB + IVM	ALB	IVM	ALB + IVM	ALB	IVM	*Trichuris trichiura*	Hookworm		*Ascaris lumbricoides*	
Bleach et al	5 weeks	7.4 years			446			121.4	112.7	120.2	21.8	22.6	22.5	N/d				
Ismail et al^[18]^	3 weeks	N/d			84			N/d			N/d			N/d				
Belizario et al^[17]^	360 days	N/d			N/d			N/d			N/d			Light: 61/58/51 Moderate: 68/74/80 Heavy: 20/17/23		N/d		Light: 33/35/31 Moderate: 42/50/43 Heavy: 30/14/28
Ndyomugyenyi et al^[21]^	21 days	23.7 (6.0)	24.0 (6.3)	23.1 (5.2)	146	140	148	156.7	156.3	156.0	56.7	56.7	56.1	N/d				
Knopp et al^[19]^	3 weeks	11.0 (2.8)	10.9 (2.6)	N/d	69	79	N/d	N/d			30.6	30.5	N/d	Light: 142/138 Moderate: 11/12 Heavy: 0/0	Light: 36/44 Moderate: 1/0 Heavy: 0/0		Light: 10/11 Moderate: 4/7 Heavy: 1/1	
Matamoros et al^[20]^	3 days	8.1 (2.5)	8.8 (2.9)	N/d	15	12	N/d	N/d			N/d			N/d				
Hürlimann et al (1)^[15]^	14–21 days	16.0 (13.4)	16.5 (14.1)	N/d	126	136	N/d	135.8	136.7	N/d	37.2	37.5	N/d	Light: 192/190 Moderate: 60/64 Heavy: 3/2	Light: 16/31 Moderate: 0/0 Heavy: 2/0		Light: 42/38 Moderate: 39/40 Heavy: 10/13	
Hürlimann et al (2)^[15]^	14–21 days	25.9 (17.4)	27.7 (17.3)	N/d	128	130	N/d	142.3	144.3	N/d	39.7	41.1	N/d	Light: 232/232 Moderate: 42/42 Heavy: 4/7	Light: 184/182 Moderate: 43/37 Heavy: 26/31		Light: 48/58 Moderate: 44/47 Heavy: 4/7	
Hürlimann et al (3)^[15]^	14–21 days	13.9 (9.6)	14.0 (10.5)	N/d	139	134	N/d	137.7	137.3	N/d	34.0	34.2	N/d	Light: 234/231 Moderate: 71/74 Heavy: 3/0	Light: 42/53 Moderate: 0/0 Heavy: 0/0		Light: 52/35 Moderate: 37/38 Heavy: 1/1	
Welsche et al^[10]^	14–21 days	15.8 (1.5)	15.6 (1.2)	15.9 (1.3)	48	6	3	157.2	159.1	158.9	47.7	50.8	48.1	Light: 148/14 Moderate: 63/5 Heavy: 0/0	Light: 70/3 Moderate: 0/0 Heavy: 0/0		Light: 49/4 Moderate: 55/7 Heavy: 6/1	

ALB = albendazole, ALB+IVM = albendazole and ivermectin, IVM = ivermectin, n = number of participants, N/d = not defined, SD = standard deviation.

**Figure 2.**
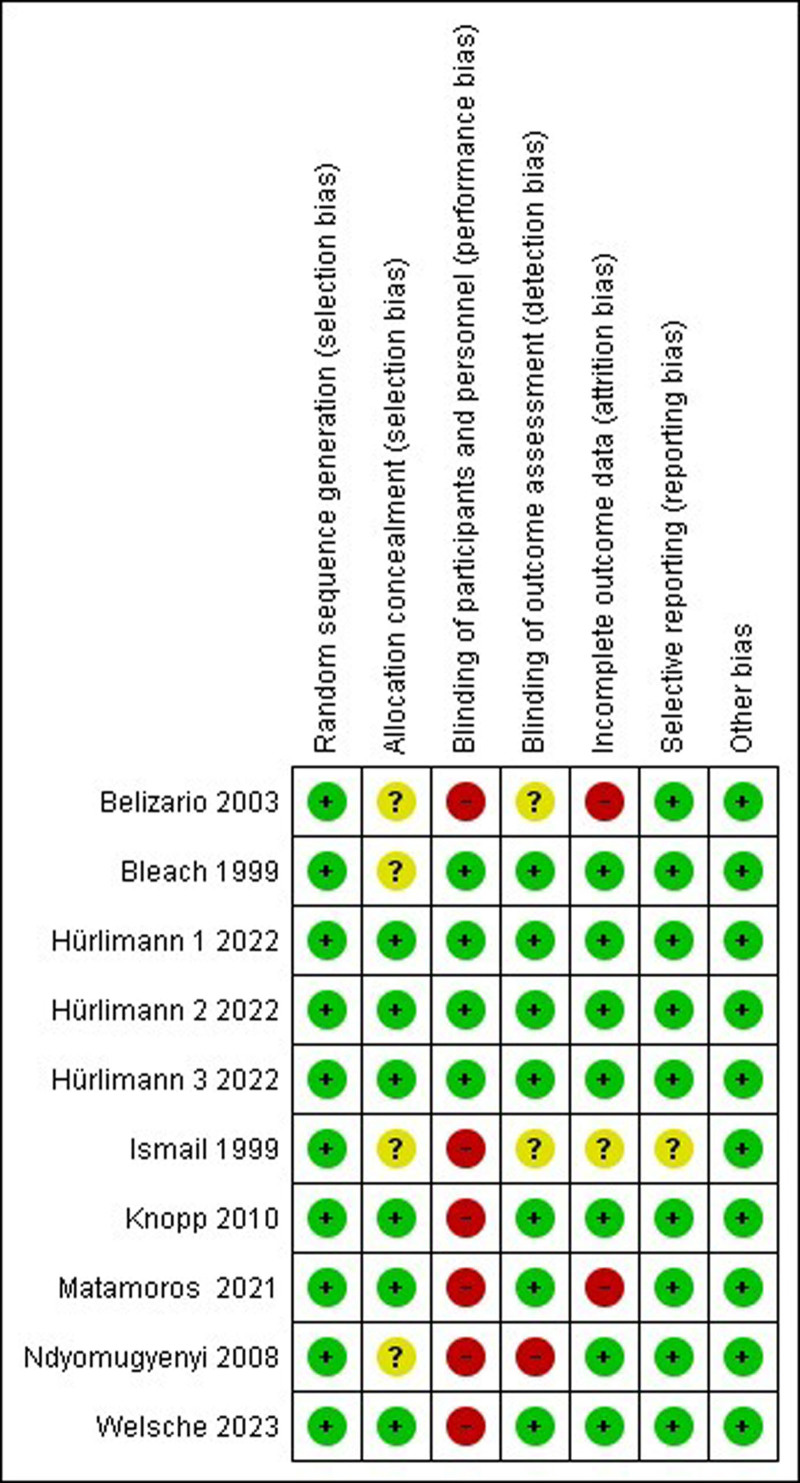
Summary of risk of bias assessments using the Cochrane risk of bias tool for randomized controlled trials.

Generally, studies were found to be of a moderate quality with an intermediate risk of bias. The majority of studies demonstrated a concerning degree of performance bias, attributed to the absent blinding of participants and personnel. Some concerns were observed with respect to detection and attrition bias. The summary of the risk of bias assessment is illustrated in Figure 2, with the risk of bias graph available in Figure S1, Supplemental Digital Content, http://links.lww.com/MD/O277.

### 3.3. Efficacy of combined therapy using ivermectin–albendazole in comparison to albendazole alone

**Figure 3.**
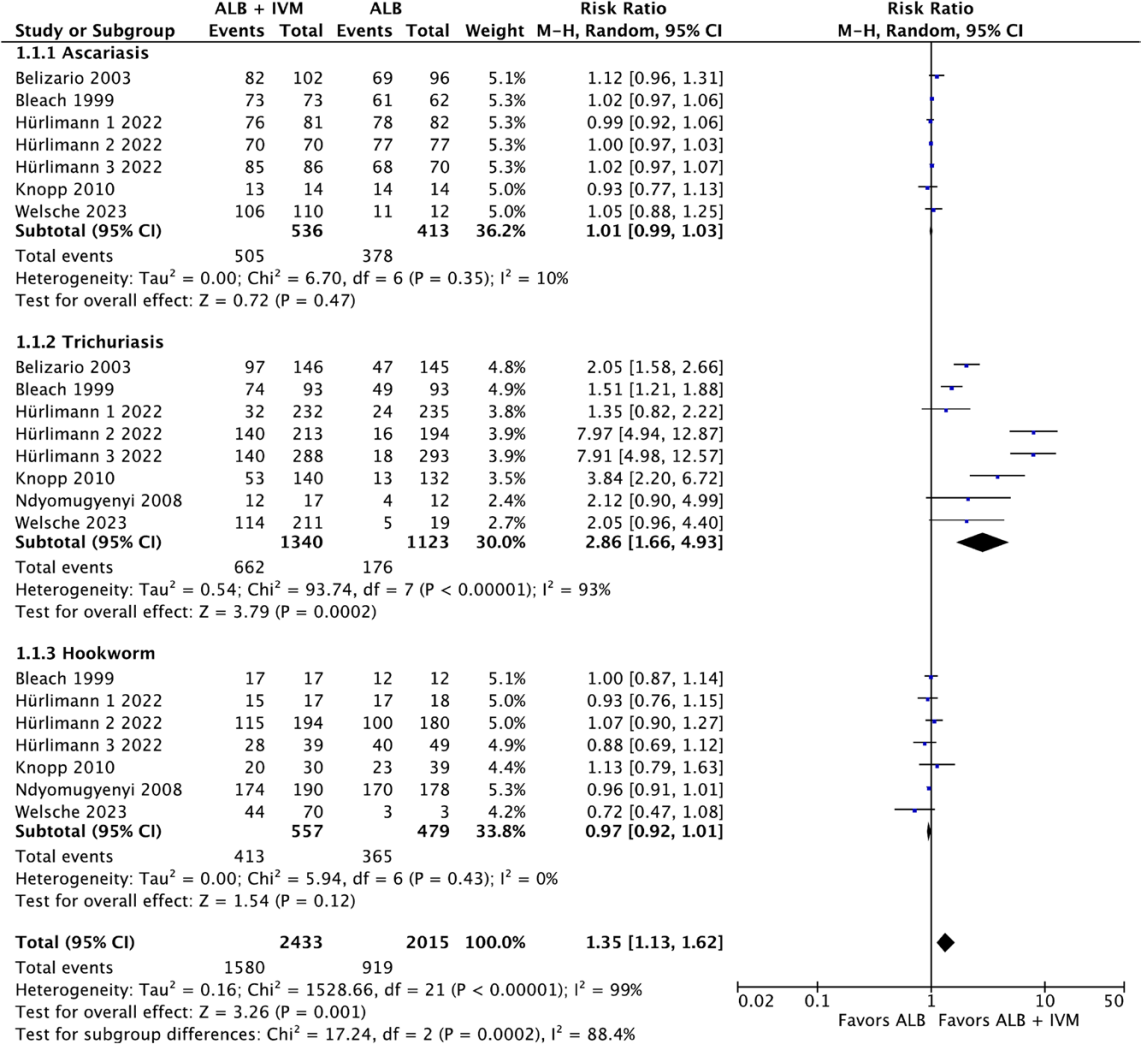
Forest plot of efficacy of ivermectin–albendazole vs albendazole-only for soil-transmitted helminths.

There were 5 studies comparing the CR of ivermectin–albendazole versus albendazole alone in ascariasis infection. The pooling of these studies revealed no statistically significant differences between the use of combined therapy versus monotherapy (RR: 1.01; 95% CI: 0.99–1.03; *P* = .47; Fig. 3). There were 6 studies comparing the CR of ivermectin–albendazole versus albendazole alone in trichuriasis infection. The aggregation of the data revealed statistically significant superiority of ivermectin–albendazole combined therapy in comparison to albendazole alone (RR: 2.86; 95% CI: 1.66–4.93; *P* = .0002; *I*^2^ = 93%; Fig. 3). There were 5 studies comparing the CR of ivermectin–albendazole versus albendazole alone in hookworm infection. The aggregation of the data revealed no statistically significant differences between the use of combined therapy versus monotherapy (RR: 0.97; 95% CI: 0.92–1.01; *P* = .12; Fig. 3). There were statistically significant subgroup differences present between the various types of STH infections (*P* = .0002).

### 3.4. Efficacy of combined therapy using ivermectin–albendazole in comparison to ivermectin alone

**Figure 4.**
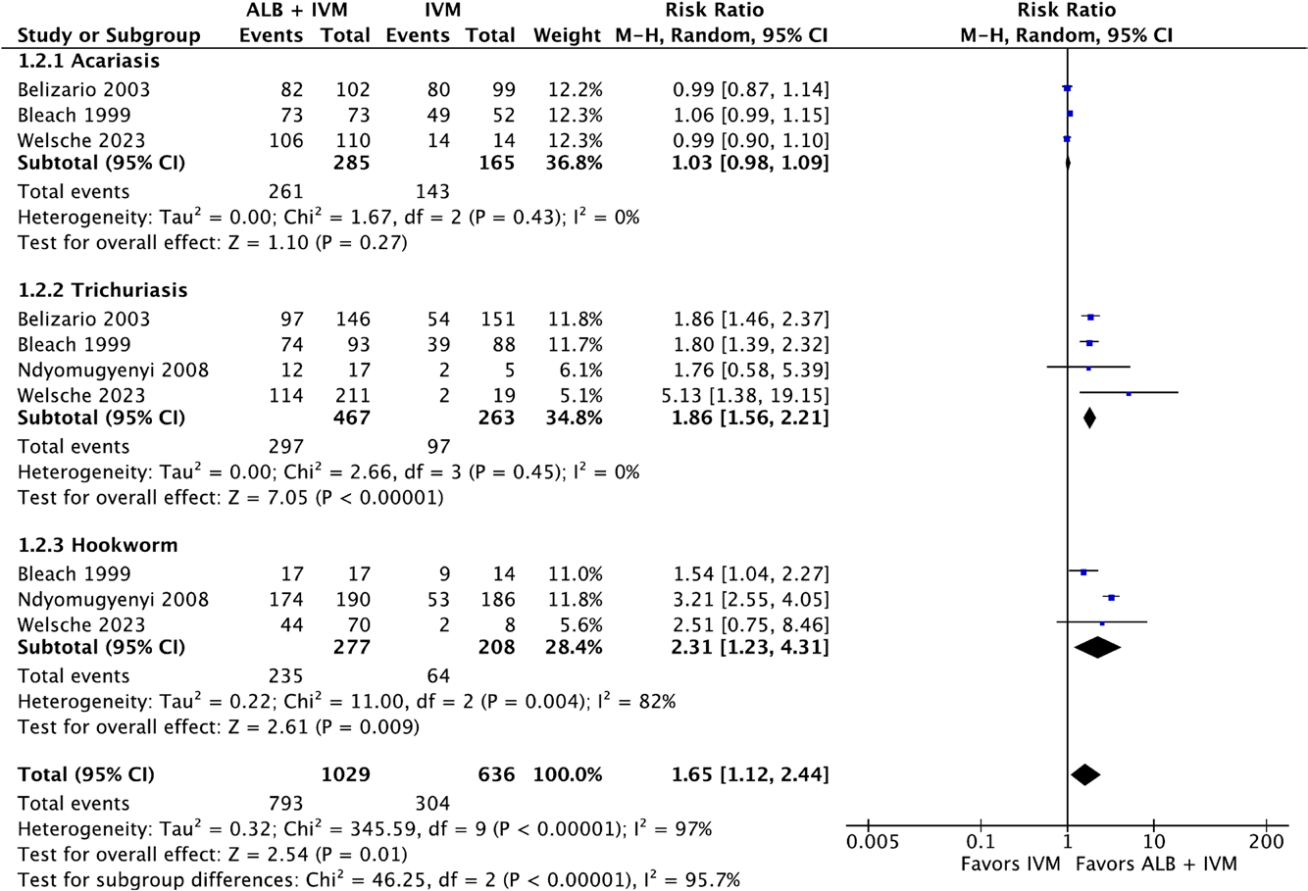
Forest plot of efficacy of ivermectin–albendazole vs ivermectin-only for soil-transmitted helminths.

There were 3 studies comparing the CR of ivermectin–albendazole versus ivermectin alone in ascariasis infection. The pooling of these studies revealed no statistically significant differences between the use of combined therapy versus monotherapy (RR: 1.03; 95% CI: 0.98–1.09; *P* = .27; Fig. 4). There were 4 studies comparing the CR of ivermectin–albendazole versus ivermectin alone in trichuriasis infection. The aggregation of the data revealed statistically significant superiority of ivermectin–albendazole in comparison to ivermectin alone (RR: 1.86; 95% CI: 1.56–2.21; *P* < .00001; Fig. 4). There were 3 studies comparing the CR of ivermectin–albendazole versus ivermectin alone in hookworm infection. The aggregation of the data revealed statistically significant superiority of ivermectin–albendazole versus ivermectin alone (RR: 2.31; 95% CI: 1.12–2.44; *P* = .01; *I*^2^ = 97%; Fig. 4). There were statistically significant subgroup differences present between the various types of STH infections (*P* < .00001).

### 3.5. Total number of patients with adverse events

**Figure 5.**
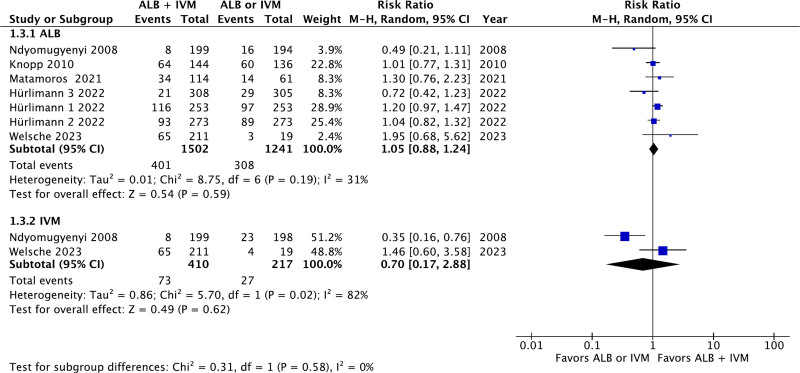
Forest plot of total number of patients with adverse events.

There were 5 studies reporting the total number of patients with adverse events within patients receiving ivermectin–albendazole versus albendazole alone. The aggregation of the data revealed no statistically significant differences between ivermectin–albendazole and albendazole alone (RR: 1.05; 95% CI: 0.88–1.24; *P* = .59; Fig. 5). Moreover, there were 2 studies reporting the total number of patients with adverse events within patients receiving ivermectin–albendazole versus ivermectin alone. The aggregation of the data revealed no statistically significant differences between ivermectin–albendazole and ivermectin alone (RR: 0.70; 95% CI: 0.17–2.88; *P* = .62; *I*^2^ = 82%; Fig. 5).

### 3.6. Incidence of abdominal pain, fever, and allergic reaction

**Figure 6.**
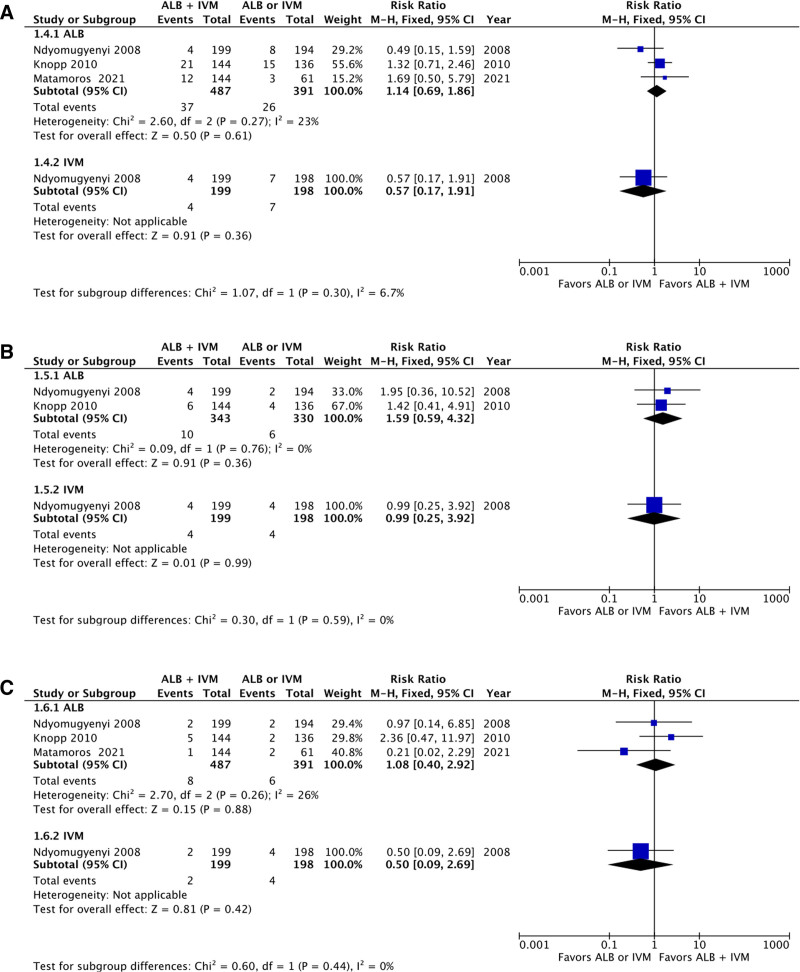
Forest plot of adverse events, including (A) abdominal pain, (B) fever, and (C) allergic reaction.

There were 3 studies reporting the incidence of abdominal pain within patients receiving combined therapy using ivermectin–albendazole versus albendazole alone. The aggregation of the data revealed no statistically significant differences between combined therapy group in comparison to the monotherapy group (RR: 1.14; 95% CI: 0.69–1.86; *P* = .61; Fig. 6A). A single study reported the incidence of abdominal pain within patients receiving combined therapy using ivermectin–albendazole versus ivermectin alone, showing no statistically significant differences between combined therapy group in comparison to the monotherapy group (RR: 0.57; 95% CI: 0.17–1.91; *P* = .36; Fig. 6A). No significant subgroup differences were detected between the albendazole-only or ivermectin-only cohorts (*P* = .30). There were 2 studies reporting the incidence of fever within patients receiving combined therapy using ivermectin–albendazole versus albendazole alone. The aggregation of the data revealed no statistically significant differences between combined therapy group in comparison to the monotherapy group (RR: 1.59; 95% CI: 0.59–4.32; *P* = .36; Fig. 6B). A single study reported the incidence of fever within patients receiving combined therapy using ivermectin–albendazole versus ivermectin alone, showing no statistically significant differences between combined therapy group in comparison to the monotherapy group (RR: 0.99; 95% CI: 0.25–3.92; *P* = .99; Fig. 6B). No significant subgroup differences were detected between the albendazole-only or ivermectin-only cohorts (*P* = .59). There were 3 studies reporting the incidence of an allergic reaction within patients receiving combined therapy using ivermectin–albendazole versus albendazole or ivermectin alone. The aggregation of the data revealed no statistically significant differences between combined therapy group in comparison to the monotherapy group (RR: 1.08; 95% CI: 0.40–2.92; *P* = .88; Fig. 6C). A single study reported the incidence of abdominal pain within patients receiving combined therapy using ivermectin–albendazole versus ivermectin alone, showing no statistically significant differences between combined therapy group in comparison to the monotherapy group (RR: 0.50; 95% CI: 0.09–2.69; *P* = .42; Fig. 6C). No significant subgroup differences were detected between the albendazole-only or ivermectin-only cohorts (*P* = .44).

### 3.7. Incidence of headache, vomiting, and nausea

**Figure 7.**
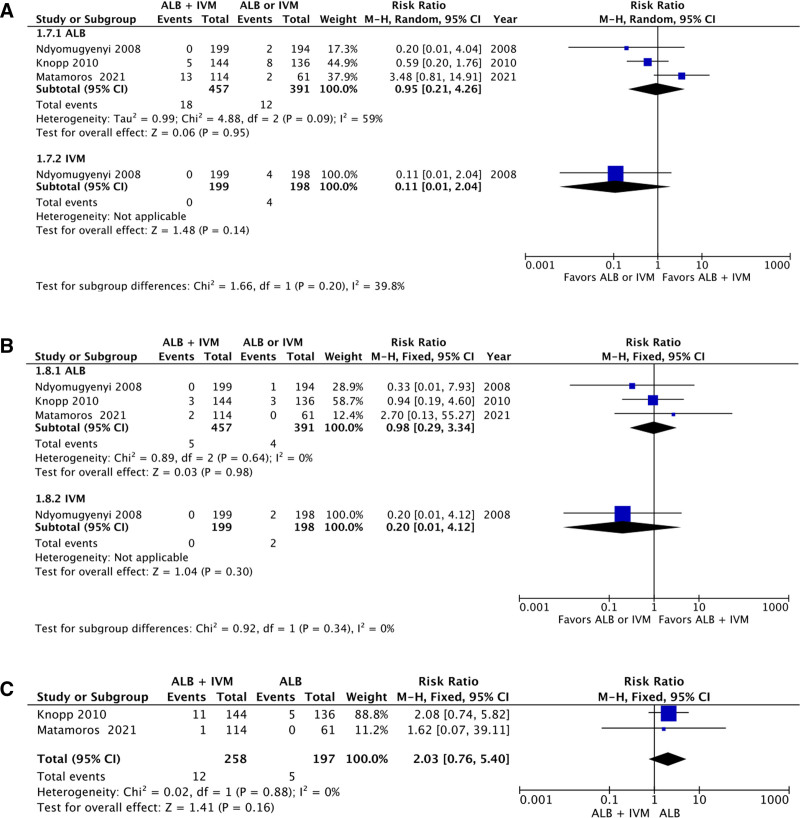
Forest plot of adverse events, including (A) headache, (B) vomiting, and (C) nausea.

There were 3 studies reporting the incidence of headache within patients receiving combined therapy using ivermectin–albendazole versus albendazole alone. The aggregation of the data revealed no statistically significant differences between combined therapy group in comparison to the monotherapy group (RR: 0.95; 95% CI: 0.21–4.26; *P* = .95; Fig. 7A). A single study reported the incidence of headache within patients receiving combined therapy using ivermectin–albendazole versus ivermectin alone, showing no statistically significant differences between combined therapy group in comparison to the monotherapy group (RR: 0.11; 95% CI: 0.01–2.04; *P* = .14; Fig. 7A). No significant subgroup differences were detected between the albendazole-only or ivermectin-only cohorts (*P* = .20). There were 3 studies reporting the incidence of vomiting within patients receiving combined therapy using ivermectin–albendazole versus albendazole alone. The aggregation of the data revealed no statistically significant differences between combined therapy group in comparison to the monotherapy group (RR: 0.98; 95% CI: 0.29–3.34; *P* = .98; Fig. 7B). A single study reported the incidence of vomiting within patients receiving combined therapy using ivermectin–albendazole versus ivermectin alone, showing no statistically significant differences between combined therapy group in comparison to the monotherapy group (RR: 0.20; 95% CI: 0.01–4.12; *P* = .30; Fig. 7B). No significant subgroup differences were detected between the albendazole-only or ivermectin-only cohorts (*P* = .34). There were 2 studies reporting the incidence of nausea within patients receiving combined therapy using ivermectin–albendazole versus albendazole or ivermectin alone. The aggregation of the data revealed no statistically significant differences between combined therapy group in comparison to the monotherapy group (RR: 2.03; 95% CI: 0.76–5.41; *P* = .16; Fig. 7C).

### 3.8. Egg reduction rate

There were 6 studies comparing the efficacy of co-administered ivermectin–albendazole versus albendazole alone in the treatment of *A lumbricoides* infection. The pooled analysis revealed a remarkable mean ERR of 100% (n = 77) in the ivermectin–albendazole arm, indicating complete eradication of *A lumbricoides* eggs in the majority of treated individuals. In contrast, the albendazole arm achieved a slightly lower mean ERR of 98.8% (n = 63), suggesting a marginally reduced efficacy compared to the combination therapy. For hookworm infection, data from 5 studies were synthesized to assess the comparative efficacy of ivermectin–albendazole versus albendazole alone. The ivemrectin–albendazole arm demonstrated a robust mean ERR of 97.7% (n = 70), whereas the albendazole arm exhibited a slightly lower mean ERR of 96.8% (n = 60), indicating comparable efficacy between the 2 treatment regimens. In trichuriasis infection, 8 studies were included for analysis. The ivermectin–albendazole arm exhibited a substantial mean ERR of 93.2% (n = 163), highlighting its efficacy in reducing *T trichiura* egg counts. In contrast, the albendazole arm showed a notably lower mean ERR of 61% (n = 137), underscoring the superior efficacy of ivermectin–albendazole in the treatment of *T trichiura* infection compared to albendazole alone. The complete details regarding ERR are available in Table 2.

**Table 2:** Summary results of egg reduction rate (ERR) of the included studies.

Studies	*Ascaris lumbricoides*, % (n)	Hookworm, % (n)	*Trichuris trichiura*, % (n)
Ismail et al^[18]^			
*IVM + ALB*	–	–	94 (53)
*ALB*	–	–	70 (55)
Belizario et al^[17]^			
*IVM + ALB*	100 (102)	–	98 (146)
*ALB*	93 (96)	–	54 (145)
Knopp et al^[19]^			
*IVM + ALB*	100 (14)	96 (30)	91.1 (140)
*ALB*	100 (14)	94 (39)	40.3 (132)
Matamoros et al^[20]^			
*IVM + ALB*	–	–	96.7 (24)
*ALB*	–	–	47.7 (24)
Hürlimann et al (1)^[15]^			
*IVM + ALB*	100 (81)	99 (17)	70 (232)
*ALB*	100 (82)	100 (28)	64 (235)
Hürlimann et al (2)^[15]^			
*IVM + ALB*	100 (70)	99 (194)	99 (213)
*ALB*	100 (77)	91 (180)	69 (194)
Hürlimann et al (3)^[15]^			
*IVM + ALB*	100 (86)	97 (39)	98 (288)
*ALB*	100 (97)	99 (49)	57 (293)
Welsche et al^[10]^			
*IVM + ALB*	100 (110)	97.4 (70)	99 (211)
*ALB*	100 (12)	100 (3)	86.2 (19)
Mean ERR			
*IVM + ALB % (n*)	100 (77)	97.7 (70)	93.2 (163)
*ALB % (n*)	98.8 (63)	96.8 (60)	61 (137)

ALB = albendazole, ERR = egg reduction rate, IVM = ivermectin, IVM + ALB = ivermectin + albendazole.

## 4. Discussion

STH infections are a global concern, affecting an estimated 1.5 billion people, particularly those in impoverished and disadvantaged communities. The primary control measures involve the use of preventive chemotherapy compounds such as albendazole and mebendazole.^[1]^ However, the success of such strategies is threatened by 2 major restrictions: the low efficacy of current anthelmintics against certain helminthes, and the potential for anthelmintic drug resistance.^[22]^ Treating *T trichiura* remains a significant challenge, as these drugs show limited efficacy against these helminthes.^[6,23]^ Therefore, more effective interventions are essential. The objective of the study was to assess the safety and efficacy of combination treatment as a potential intervention for clinical practice and public health strategies targeting the control of STH.

Our meta-study revealed that for *T trichiura* infections, the co-administration of ivermectin–albendazole resulted in significantly higher CRs compared to individual drug treatments with albendazole or ivermectin only, with RR of 2.86 and 1.86, respectively. This suggests that combination therapy may offer a more effective approach for treating *T trichiura* infections compared to conventional single-drug treatments.^[8,24]^ However, for *A lumbricoides* species, our analysis indicated that ivermectin–albendazole produced comparable CRs to individual drug treatments, implying that single-dose albendazole is equally effective at curing *A lumbricoides* infections as combination treatment with ivermectin–albendazole. Similarly, for hookworm infections, ivermectin–albendazole showed comparable CRs with single-dose albendazole, while ivermectin-only treatment was found to be inferior to the combination therapy in terms of CRs. These findings suggest that ivermectin–albendazole is either equally or less efficacious compared to single-dose albendazole for curing *A. lumbricoides* and hookworm infections. However, the addition of ivermectin to conventional treatment with single dose albendazole may enhance *T trichiura* control, the most challenging STH to manage, without compromising efficacy against other STH infections. Similar findings are reported in previous literature.^[8,23,24]^

Comparing the second efficacy outcome of mean ERR between co-administered ivermectin–albendazole and albendazole alone for treating *A lumbricoides* and hookworm infections yielded compelling results. Remarkably, in the ivermectin–albendazole arm, ERR reached an impressive 100% and 97.7%, respectively, indicating the complete eradication of *A lumbricoides* eggs in the majority of treated individuals. In contrast, the albendazole arm achieved a slightly lower mean ERR of 98.8% and 96.8%, reflecting a marginally reduced efficacy compared to the combination therapy. While comparable ERRs were observed between the 2 treatment arms, the slightly superior efficacy of co-administered ivermectin–albendazole is underscored by its ability to achieve complete egg clearance, a clinical outcome with significant implications for reducing the risk of reinfection and transmission of *A lumbricoides* and hookworm. These findings align with previous research^[8]^ suggesting a synergistic effect of ivermectin and albendazole in helminthic infections and highlight the potential of combination therapy as a highly effective treatment option for STH. Such insights hold implications for guiding clinical practice and public health interventions in areas endemic to *A lumbricoides*, hookworms, and other STH infections.

The literature consistently supports the notion that combining ivermectin with albendazole yields either comparable or superior efficacy compared to benzimidazoles alone.^[9]^ This corroborates the findings of our study. Notably, conventional treatments such as albendazole and mebendazole have shown limited efficacy in eradicating *T trichiura* infections and reducing worm burden.^[6,25]^ Moreover, concerns regarding potential drug resistance have garnered increasing attention in recent discourse.^[26]^ The exceptional long-term effectiveness of ivermectin–albendazole can be attributed to its initial robust performance, which endures even after 1 year of treatment despite the risk of reinfection. Remarkably, in Lao PDR, we observed a substantial decrease in *T trichiura* prevalence from 100.0% to 23.0% over the 12-month study period. Furthermore, this combination therapy resulted in a significant reduction in moderate and heavy *T trichiura* infections, a crucial public health metric,^[27]^ achieving infection rates below 1.5% within the 12-month timeframe in both countries. These findings are in line with the WHO’s ambitious target of eliminating STH infections by 2030.^[28]^

Notably, a recent network meta-analysis reported that the efficacy of 2 commonly used anthelmintics (albendazole and mebendazole) might be decreasing over time, which might be the result of emerging drug resistance to anthelmintic drugs.^[24]^ As preventative chemotherapy has expanded in recent years, there has been a notable rise in selective drug pressure on populations of STH. This escalation has the potential to incite the development of anthelmintic drug resistance.^[29]^ However, evidence for such resistance in humans is scant due to a lack of relevant studies. Nevertheless, frequent and rapid resistance selection against major anthelmintics is a well-documented phenomenon in veterinary medicine.^[30]^ The reduced efficacy of currently widely used drugs for STH and the emerging challenge of anthelmintic drug resistance make new interventions a pressing need. Co-administration of drugs offers the advantage of increased efficacy by acting on different targets compared to monotherapy. The combination of drugs is also helpful in delaying the emergence of drug resistance. This long-term benefit is expected to outweigh the associated increase in treatment costs.^[22]^

Regarding safety, the co-administration of ivermectin and albendazole for treating STH is generally safe. The adverse events were mild and transient, and the number of people experiencing adverse effects was not different in co-treated and single drug-treated patients. Our study assessed safety data from a range of populations, including preschool and school-aged children, adult men, and pregnant women, and found the co-administration generally well-tolerated.^[10,15,17–19,21]^ Pharmacokinetic studies in healthy humans also showed that co-administration of ivermectin and albendazole does not increase their toxicity or affect absorption, metabolism, or excretion.^[31]^ The safety profile of combination treatment has already been reviewed by a study on lymphatic filariasis.^[32]^ The study concluded that combination treatment did not increase the frequency or intensity of adverse events, even with the most rigorous methods of monitoring, including hematological and biomedical laboratory parameters.^[32]^ Albendazole and ivermectin are already being used on a wide scale for lymphatic filiarasis control programs^[33]^ since their addition in WHO’s list of essential medicines for STH control^[34]^ and have a well-established safety profile.^[8,32]^

STH infections pose a significant public health challenge in resource-limited settings because of their capacity to sustain poverty. Persistent infections are linked to adverse impacts on children’s learning and growth, as well as diminished productivity in adults.^[1]^ The addition of ivermectin to albendazole can have a significant impact on reducing the burden of these infections, fighting the emerging challenges of drug resistance that is reducing the efficacy of these drugs against *T trichiura*. The inclusion of ivermectin–albendazole co-treatment in the WHO Model List of Essential Medicines is indeed a significant step towards improving the control of STH infections. Inclusion on the WHO’s essential medicines list signifies that these medications should be consistently available in healthcare systems in suitable dosage forms, ensuring quality reassurance and affordable prices for the healthcare system.^[34]^ The additional benefits of co-administration are beneficial for the control of diseases like strongyloid stercoralis, scabies, and lymphatic filariasis.^[35–37]^ However, it is reported that individuals co-infected with Loa Loa, a filarial nematode, experience more adverse events when treated with ivermectin. Therefore, it is advised that individuals be screened in Loa Loa endemic areas before rolling out treatment with ivermectin.^[38]^

### 4.1. Future recommendations

Numerous unexplored research avenues warrant attention. Investigating the most effective dosing regimens and treatment protocols for the combined use of ivermectin and albendazole presents an opportunity to optimize treatment outcomes by examining dosage variations and treatment duration. Targeted subgroup analyses focusing on distinct demographic cohorts, including children, pregnant women, and immunocompromised individuals, offer the potential for tailored therapeutic insights. Longitudinal studies are essential to ascertain the sustained efficacy and safety of the combined therapy vis-à-vis monotherapy over extended periods. Delving into potential resistance emergence to ivermectin, albendazole, or their combination is imperative to elucidate resistance mechanisms, guiding preemptive measures against or mitigation of resistance risks. Economic evaluations comparing the cost-effectiveness of co-administered therapy with monotherapy across diverse healthcare settings are vital for informed resource allocation by healthcare decision-makers. Exploring synergistic combinations of ivermectin and albendazole with other anthelmintic agents or adjunct therapies could significantly amplify treatment efficacy while countering potential drug resistance. Conducting focused clinical trials targeting specific helminth species promises nuanced insights into tailored treatment modalities for each parasite. Additionally, real-world effectiveness studies spanning diverse geographical regions and healthcare settings serve to authenticate observations gleaned from controlled trial environments.

### 4.2. Limitations

Our comprehensive analysis investigated the comparative effectiveness and safety of simultaneously administered ivermectin and albendazole in contrast to singular therapy for STH. Despite our rigorous approach, certain limitations require Acknowledgments. The availability of randomized controlled trial exploring this co-administration approach against a singular approach was notably low, thus resulting in a limited sample size. This constraint, combined with substantial heterogeneity in methodologies and participant demographics across the included studies, poses challenges to the depth and precision of our assessment. The wide range of treatment durations utilized among the included study populations pose as a potential source of heterogeneity, paired with the inability to effective perform subgroup analyses on such basis serves as a notable limitation of our analysis. The absence of prolonged follow-up in a significant portion of studies limits our ability to comprehensively evaluate sustained treatment efficacy and safety, relying primarily on short-term outcomes that may skew our analysis. Additionally, incomplete reporting of adverse events hindered our ability to thoroughly assess treatment safety in addition to efficacy. Geographical variations, parasite resistance patterns, and individual patient characteristics also likely influence the efficacy and safety of the co-administered therapy, potentially constraining its applicability to diverse global populations in varying contexts.

## 5. Conclusion

The systematic investigation through a comprehensive review and meta-analysis delved into the comparative effectiveness and safety of concurrently administered ivermectin and albendazole versus singular therapy for the treatment of soil-transmitted helminths. The evaluation across diverse helminth infections revealed varied outcomes in terms of efficacy. While no substantial distinction was discerned between combined therapy and monotherapy in ascariasis and hookworm infections, a notable superiority emerged in the treatment of trichuriasis with the combined approach. Importantly, when juxtaposed with ivermectin monotherapy, the combined therapy exhibited noteworthy advantages in addressing trichuriasis and hookworm infections. Noteworthy is the absence of significant disparities in adverse events between the combined therapy and monotherapy groups, indicating comparable safety profiles. These findings highlight the nuanced effectiveness of combined therapy specific to helminth types and underscore its favorable safety profile, thereby providing pivotal insights that contribute to the evolving landscape of soil-transmitted helminth treatment strategies.

## Author contributions

**Conceptualization:** Muhammad Fazal Ud Din, Maham Khalid.

**Data curation:** Hurais Malik, Muhammad Aqib Faizan, Tooba Rehman, Syed Muhammad Abdullah Shah.

**Formal analysis:** Hurais Malik, Muhammad Aqib Faizan, Tooba Rehman, Syed Muhammad Abdullah Shah, Syeda Fatima Amir, Musarrat Fraz.

**Methodology:** Hurais Malik, Muhammad Hudaib.

**Supervision:** Muhammad Umair Anjum, Muhammad Omar Larik, Hurais Malik, Pratik Bhattarai.

**Writing – original draft:** Hurais Malik, Muhammad Fazal Ud Din, Muhammad Hudaib, Abdullah Abid Khan, Maham Khalid.

**Writing – review & editing:** Hurais Malik, Muhammad Umair Anjum, Muhammad Omar Larik, Pratik Bhattarai.

## Supplementary Material


