# Supplementary material for: Efficacy and safety of ivermectin–albendazole combination versus ivermectin or albendazole monotherapy in soil-transmitted helminthiasis: A systematic review and meta-analysis
Source: Medicine (Baltimore). 2025 Jan 17;104(3):e41280. doi: 10.1097/MD.0000000000041280 (PMC11749739; doi:10.1097/MD.0000000000041280)
Supplement: Supplementary file 1 [file medi-104-e41280-s001.docx]

**Supplementary Table S1.** Detailed search strategies employed for each database.

| **Databases** | **Search Strings** |
| --- | --- |
| MEDLINE | "soil-transmitted helminths" OR "STH" OR "soil transmitted helminths" OR "Helminths" OR "Helminth" OR "Worms, Parasitic" OR "Parasitic Worms" OR "Parasitic Worm" OR "Worm, Parasitic" OR "Aschelminthes" OR "Aschelminthe" OR "Nematomorpha" OR "Nematomorphas" OR "Gordius" OR "Ascaris lumbricoides" OR "Ascaris lumbricoide" OR "lumbricoides, Ascaris" OR "Whipworm Infections" OR "Whipworm" OR "Infections, Whipworm" OR "Infection, Whipworm" OR "Whipworm Infection" OR "Trichuris Infections" OR "Infections, Trichuris" OR "Infection, Trichuris" OR "Trichuris Infection" OR "Trichuris trichiura Infections" OR "Infections, Trichuris trichiura" OR "Infection, Trichuris trichiura" OR "Trichuris trichiura Infection" OR "Trichocephaliases" OR "Trichocephaliasis" OR "Trichuriases" OR "Trichuriasis" OR "trichiura, Trichuris" OR "Trichuris trichiuras" OR "Trichuris trichiura" OR "Trichocephalus" OR "Trichuri" OR "Trichuris" OR "Hookworm Infections" OR "Infections, Hookworm" OR "Hookworm Infection" OR "Infection, Hookworm" OR "Bunostomiasis" OR "Bunostomiases" AND "Ivomec" OR "Eqvalan" OR "MK933" OR "MK 933" OR "MK-933" OR "Mectizan" OR "Stromectol" OR "Ivermectin" AND "SKF62979" OR "SKF 62979" OR "SKF-62979" OR "SK and F62979" OR "SK and F 62979" OR "SK and F-62979" OR "Gascop" OR "Eskazole" OR "Endoplus" OR "Mediamix V Disthelm" OR "Disthelm" OR "Digezanol" OR "Bendapar" OR "Monohydrochloride, Albendazole" OR "Albendazole Monohydrochloride" OR "Albendoral" OR "Zentel" OR "Albenza" OR "Andazol" OR "Metiazol" OR "Bilutac" OR "Lurdex" OR "Albendazole" |
| CENTRAL | "soil-transmitted helminths" OR "STH" OR "soil transmitted helminths" OR "Helminths" OR "Helminth" OR "Worms, Parasitic" OR "Parasitic Worms" OR "Parasitic Worm" OR "Worm, Parasitic" OR "Aschelminthes" OR "Aschelminthe" OR "Nematomorpha" OR "Nematomorphas" OR "Gordius" OR "Ascaris lumbricoides" OR "Ascaris lumbricoide" OR "lumbricoides, Ascaris" OR "Whipworm Infections" OR "Whipworm" OR "Infections, Whipworm" OR "Infection, Whipworm" OR "Whipworm Infection" OR "Trichuris Infections" OR "Infections, Trichuris" OR "Infection, Trichuris" OR "Trichuris Infection" OR "Trichuris trichiura Infections" OR "Infections, Trichuris trichiura" OR "Infection, Trichuris trichiura" OR "Trichuris trichiura Infection" OR "Trichocephaliases" OR "Trichocephaliasis" OR "Trichuriases" OR "Trichuriasis" OR "trichiura, Trichuris" OR "Trichuris trichiuras" OR "Trichuris trichiura" OR "Trichocephalus" OR "Trichuri" OR "Trichuris" OR "Hookworm Infections" OR "Infections, Hookworm" OR "Hookworm Infection" OR "Infection, Hookworm" OR "Bunostomiasis" OR "Bunostomiases" AND "Ivomec" OR "Eqvalan" OR "MK933" OR "MK 933" OR "MK-933" OR "Mectizan" OR "Stromectol" OR "Ivermectin" AND "SKF62979" OR "SKF 62979" OR "SKF-62979" OR "SK and F62979" OR "SK and F 62979" OR "SK and F-62979" OR "Gascop" OR "Eskazole" OR "Endoplus" OR "Mediamix V Disthelm" OR "Disthelm" OR "Digezanol" OR "Bendapar" OR "Monohydrochloride, Albendazole" OR "Albendazole Monohydrochloride" OR "Albendoral" OR "Zentel" OR "Albenza" OR "Andazol" OR "Metiazol" OR "Bilutac" OR "Lurdex" OR "Albendazole" |
| Google Scholar | "soil-transmitted helminths" OR "STH" OR "soil transmitted helminths" OR "Helminths" OR "Helminth" OR "Worms, Parasitic" OR "Parasitic Worms" OR "Parasitic Worm" OR "Worm, Parasitic" OR "Aschelminthes" OR "Aschelminthe" OR "Nematomorpha" OR "Nematomorphas" OR "Gordius" OR "Ascaris lumbricoides" OR "Ascaris lumbricoide" OR "lumbricoides, Ascaris" OR "Whipworm Infections" OR "Whipworm" OR "Infections, Whipworm" OR "Infection, Whipworm" OR "Whipworm Infection" OR "Trichuris Infections" OR "Infections, Trichuris" OR "Infection, Trichuris" OR "Trichuris Infection" OR "Trichuris trichiura Infections" OR "Infections, Trichuris trichiura" OR "Infection, Trichuris trichiura" OR "Trichuris trichiura Infection" OR "Trichocephaliases" OR "Trichocephaliasis" OR "Trichuriases" OR "Trichuriasis" OR "trichiura, Trichuris" OR "Trichuris trichiuras" OR "Trichuris trichiura" OR "Trichocephalus" OR "Trichuri" OR "Trichuris" OR "Hookworm Infections" OR "Infections, Hookworm" OR "Hookworm Infection" OR "Infection, Hookworm" OR "Bunostomiasis" OR "Bunostomiases" AND "Ivomec" OR "Eqvalan" OR "MK933" OR "MK 933" OR "MK-933" OR "Mectizan" OR "Stromectol" OR "Ivermectin" AND "SKF62979" OR "SKF 62979" OR "SKF-62979" OR "SK and F62979" OR "SK and F 62979" OR "SK and F-62979" OR "Gascop" OR "Eskazole" OR "Endoplus" OR "Mediamix V Disthelm" OR "Disthelm" OR "Digezanol" OR "Bendapar" OR "Monohydrochloride, Albendazole" OR "Albendazole Monohydrochloride" OR "Albendoral" OR "Zentel" OR "Albenza" OR "Andazol" OR "Metiazol" OR "Bilutac" OR "Lurdex" OR "Albendazole" |
| Embase | "soil-transmitted helminths" OR "STH" OR "soil transmitted helminths" OR "Helminths" OR "Helminth" OR "Worms, Parasitic" OR "Parasitic Worms" OR "Parasitic Worm" OR "Worm, Parasitic" OR "Aschelminthes" OR "Aschelminthe" OR "Nematomorpha" OR "Nematomorphas" OR "Gordius" OR "Ascaris lumbricoides" OR "Ascaris lumbricoide" OR "lumbricoides, Ascaris" OR "Whipworm Infections" OR "Whipworm" OR "Infections, Whipworm" OR "Infection, Whipworm" OR "Whipworm Infection" OR "Trichuris Infections" OR "Infections, Trichuris" OR "Infection, Trichuris" OR "Trichuris Infection" OR "Trichuris trichiura Infections" OR "Infections, Trichuris trichiura" OR "Infection, Trichuris trichiura" OR "Trichuris trichiura Infection" OR "Trichocephaliases" OR "Trichocephaliasis" OR "Trichuriases" OR "Trichuriasis" OR "trichiura, Trichuris" OR "Trichuris trichiuras" OR "Trichuris trichiura" OR "Trichocephalus" OR "Trichuri" OR "Trichuris" OR "Hookworm Infections" OR "Infections, Hookworm" OR "Hookworm Infection" OR "Infection, Hookworm" OR "Bunostomiasis" OR "Bunostomiases" AND "Ivomec" OR "Eqvalan" OR "MK933" OR "MK 933" OR "MK-933" OR "Mectizan" OR "Stromectol" OR "Ivermectin" AND "SKF62979" OR "SKF 62979" OR "SKF-62979" OR "SK and F62979" OR "SK and F 62979" OR "SK and F-62979" OR "Gascop" OR "Eskazole" OR "Endoplus" OR "Mediamix V Disthelm" OR "Disthelm" OR "Digezanol" OR "Bendapar" OR "Monohydrochloride, Albendazole" OR "Albendazole Monohydrochloride" OR "Albendoral" OR "Zentel" OR "Albenza" OR "Andazol" OR "Metiazol" OR "Bilutac" OR "Lurdex" OR "Albendazole" |
